# The impact of Hurricane Michael on longleaf pine habitats in Florida

**DOI:** 10.1038/s41598-020-65436-9

**Published:** 2020-05-21

**Authors:** Nicole E. Zampieri, Stephanie Pau, Daniel K. Okamoto

**Affiliations:** 1 Department of Geography, Florida State University, 113 Collegiate Loop, Tallahassee, 32306 FL USA; 2 Department of Biological Science, Florida State University, 319 Stadium Drive, Tallahassee, 32304 FL USA

**Keywords:** Forest ecology, Population dynamics, Biogeography, Climate-change ecology, Conservation biology, Forest ecology, Grassland ecology

## Abstract

Global biodiversity hotspots (GBHs) are increasingly vulnerable to human stressors such as anthropogenic climate change, which will alter the ecology of these habitats, even where protected. The longleaf pine (*Pinus palustris*) ecosystem (LPE) of the North American Coastal Plain is a GBH where disturbances are integral for ecosystem maintenance. However, stronger storms due to climate change may be outside their historical norm. In this study, we estimate the extent of Florida LPE that was directly affected by Hurricane Michael in 2018, an unprecedented Category 5 storm. We then leveraged a unique data set in a Before-After study of four sites within this region. We used variable-area transects and generalized linear mixed-effects models to estimate tree densities and logistic regression to estimate mortality by size class. We found at least 28% of the global total remaining extent of LPE was affected in Florida alone. Mortality was highest in medium sized trees (30–45 cm dbh) and ranged from 4.6–15.4% at sites further from the storm center, but increased to 87.8% near the storm center. As the frequency and intensity of extreme events increases, management plans to mitigate climate change need to account for large-scale stochastic mortality events to preserve critical habitats.

## Introduction

Ecological disturbances play an integral role in maintaining ecosystem structure and functioning^1–3^. Many ecological disturbances are expected to change with anthropogenic climate change^4^, altering the frequency, intensity, duration, and timing of events^5^. Shifting disturbance regimes due to climate change pose a threat to the conservation of biodiversity as species experience conditions outside their historical norms^6–8^. In savanna systems, which are characterized by a grassy or herbaceous understory and low tree densities, changing disturbance regimes can trigger demographic transitions altering the density of trees^9–11^, upon which biodiversity depends.

Longleaf pine (*Pinus palustris* Mill.) habitats located within the North American Coastal Plain (NACP) are a global biodiversity hotspot^12,13^. These savanna-type systems provide critical habitat for numerous endangered plant and animal species, which are dependent on the presence of sparse but critically important mature longleaf pine trees^14–17^. The canopy of longleaf pine habitats is generally monotypic, with a range of tree densities (from <100 to 300+ tree/ha), a largely open canopy, and an herbaceous, grass dominated understory^18–20^. Frequent seasonal fire is an integral part of this ecosystem and is the most important process for maintaining ecosystem structure and function^12,21–24^. The highest quality stands are dominated by mature trees with a sufficiently frequent fire interval (1–5 year return) to promote regeneration and maintain a highly biodiverse understory – containing as many as >40 species per m^2,17,25–28^. Canopy gaps are critical in promoting this biodiverse understory^29^ and allow for recruitment and regeneration of longleaf pine^30,31^. Gaps allow for greater light penetration and colonization by shade-intolerant species^32,33^. Most successful recruitment of longleaf pine requires exposed bare mineral soil and patches in the canopy to be opened up by disturbances such as fire, wind, or rain events^30,31,34^.

Florida, and more specifically the Florida Panhandle, is one of the most important strongholds of endangered longleaf pine habitat^35,36^ containing 50% and 28%, respectively, of all the remaining longleaf pine ecosystem^37–39^. Florida, and the NACP in general, borders the Gulf and Atlantic coast, and is subject to frequent storm events^12^. Thus disturbances in these regions include hurricanes and other extreme wind and rain events, in addition to fire^1,40^. Over the course of a century, the entire range of the NACP will have experienced at least one major hurricane (Category 3 and above)^32,40,41^. Numerous studies have assessed the damage to forests and savannas in the NACP after major storm events^42–46^. Species that evolved within the coastal plain, such as longleaf pines, bald cypress, and live oaks, have been shown to have lower mortality from hurricanes^44,45,47–50^ than species whose evolutionary range extends beyond the coastal plain region (such as loblolly pine or water oak), possibly due to strong selection pressure from frequent exposure to high wind storms^44^. However, longleaf pines grow at low densities and are highly susceptible to wind flow in extreme wind events^51^. In addition, as the climate changes, high wind storm events such as hurricanes and tornadoes will increase in strength and/or frequency, outside of the system’s historic norms^5,52–54^.

Hurricanes may contribute to gap dynamics in longleaf systems by removing older, rotten trees and other species that may compete for light and other resources with understory components^1,19,32,34,48,55^. While gap dynamics driven by typical tropical storm events, in addition to low intensity fires, play an important role in maintaining these open-canopied habitats, the potential for hurricanes of increasing strength to occur over the next century^5,52,53^ could lead to severe damage and potentially permanent losses of remnant stands of an already vulnerable system^12,35^. The loss of mature trees, creation of large canopy gaps, and severe damage to the understory from extreme events can have negative effects on numerous species that depend on mature trees, impede natural regeneration, alter the fire regime, increase the chance of invasive species establishment, and provide favorable conditions for insect outbreaks^4,5,56–60^.

**Figure 1:**
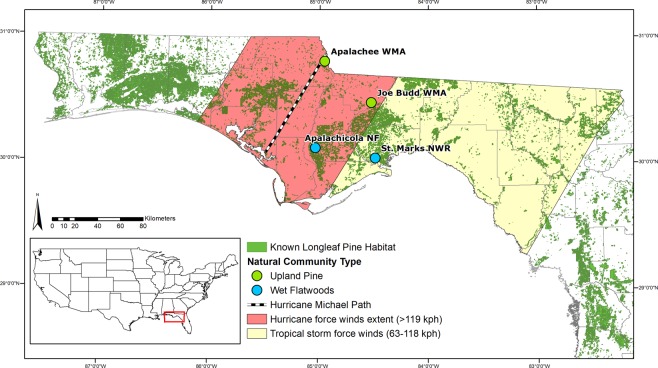
Map of study sites and storm coverage. Hurricane Michael made landfall as a Category 5 hurricane on October 10th, 2018 near Mexico Beach, FL, USA. Maximum sustained winds at landfall were approximately 257 km/h and minimum barometric pressure was 919 mb. Hurricane-force winds extended outward from the center up to 75 km and tropical storm force winds extended outward up to 280 km^61,62^. The four study sites in the Florida Panhandle in the path of Hurricane Michael include: Apalachee WMA, Joe Budd WMA, Apalachicola NF, and St. Marks NWR. The “known” longleaf pine habitat is extracted from the LPEGDB^39^. Map created in ArcMap 10.6.1^86^.

On October 10^th^, 2018 Hurricane Michael made landfall in the Florida Panhandle as the first Category 5 storm on modern record in the region. It was the strongest hurricane to make landfall in the continental U.S. since Hurricane Andrew in 1992, with maximum sustained winds of 257 km/h and minimum barometric pressure of 919 mb^61,62^. This study takes advantage of four sites of longleaf pine habitat within the path of Hurricane Michael, which were fortuitously censused during the summer before the hurricane and occur over a gradient of hurricane impact (Fig. 1). These sites were measured again after the hurricane to quantify a range of hurricane effects on longleaf forests. The objectives of this study were to (1) determine the extent of longleaf pine habitat in Florida impacted by Hurricane Michael using Geographic Information Systems (GIS) and the Longleaf Pine Ecosystem Geodatabase (LPEGDB)^39^, (2) perform Before-After field surveys of tree density and estimate mortality by size class (juvenile, small-mature, medium-mature, and large-mature) at four sites within the path of the storm using generalized linear models, and (3) compare damage types (uprooted, snapped, crown damage) at these sites, which occur in different community types that differ in their soils, hydrology, and species composition. We discuss implications of increasingly strong and frequent storms for management and restoration of this global biodiversity hotspot.

## Results

### Extent of longleaf pine habitat impacted by hurricane michael

Within the Florida Panhandle, tropical storm force winds (280 km buffer^61,62^) generated by the storm impacted at least 533,000 ha of “known” longleaf pine habitat, i.e., habitat that has been confirmed through field surveys according to the LPEGDB^39^. As much as 1,043,000 ha may possibly have been affected when including “known”, as well as “expected” (15,000 ha) and “potential” habitat (495,000 ha; see Methods). Hurricane force winds (75 km buffer^61,62^) occurred over 114,000 ha of “known” longleaf pine habitat. An additional 4,000 ha of “expected” and an additional 54,000 ha of “potential” habitat were also within the hurricane force winds buffer, for a possible total of 172,000 ha. The areas affected by hurricane force winds include large areas of managed lands with critical remaining habitat for longleaf and other threatened habitats, including the Apalachicola National Forest and Torreya State Park, as well as smaller, more isolated managed areas such as Apalachicola Bluffs and Ravines Preserve and Three Rivers State Park. The areas affected by tropical storm force winds extend further into the Panhandle and north-central Florida and include critical management areas such as Eglin Air Force Base and Blackwater River State Forest to the west and Osceola National Forest to the east.

### Changes in longleaf densities pre- and post-hurricane and estimates of mortality

**Figure 2:**
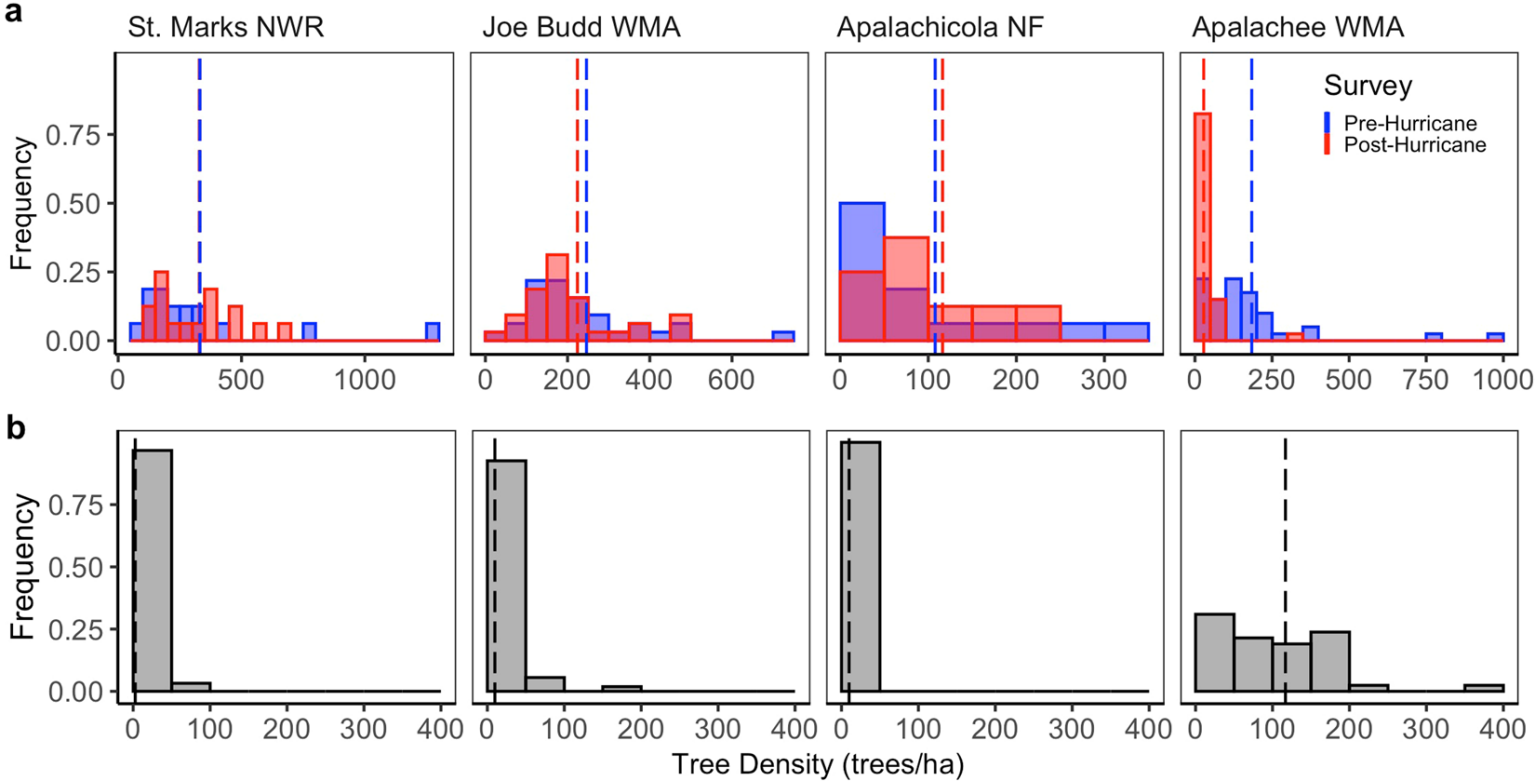
Pre- and post-hurricane living and dead tree density frequency histogram. (**a**) Histograms of pre- and post- hurricane living tree densities from each cell in all plots show the most dramatic change in tree density at Apalachee WMA, whereas other sites show less change or no detectable change. Group means of living tree density are indicated by dashed lines. Each site is scaled on a different x-axis for clearer visualization. Sites are listed in order of decreasing distance to the storm center. (**b**) Histograms of dead tree densities from each cell in all plots at all sites post-hurricane. The mean overall dead tree densities are indicated by dashed lines.

**Figure 3:**
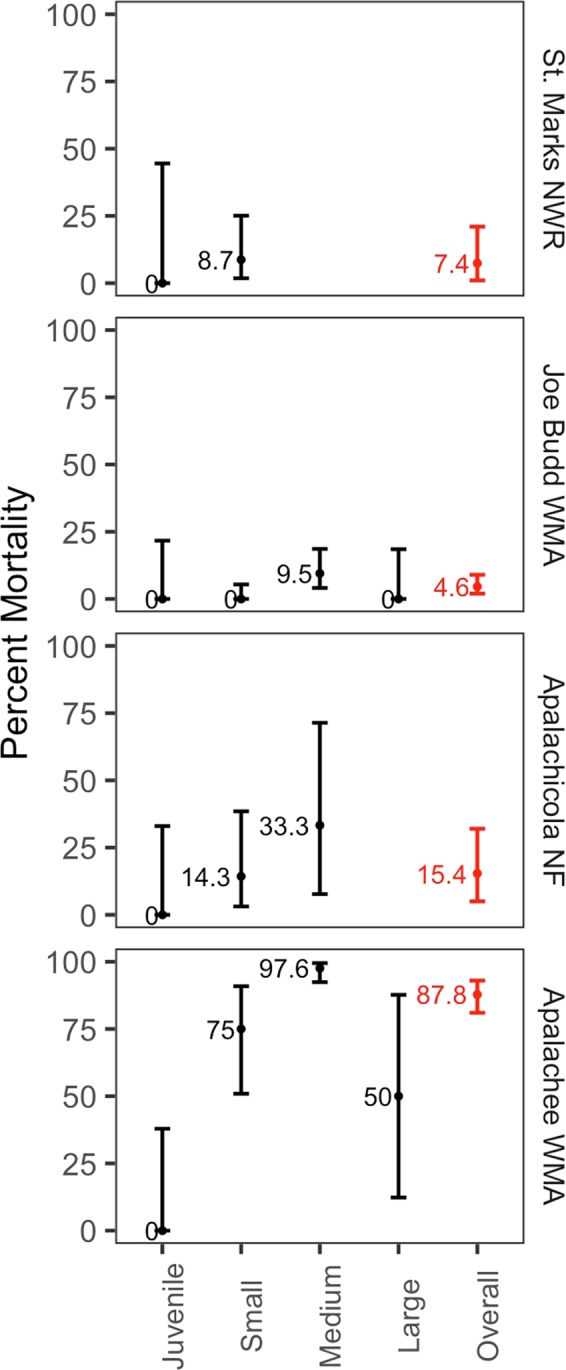
Estimated longleaf pine mortality by size class. We estimated site level mortality (overall and by size class) using logistic regression. Estimated mean percent mortality value for each size class and overall is indicated on the plots. Overall percent mortality at each site is in red. 95% confidence intervals are presented in the error bars. Sites are listed in order of decreasing distance to the storm center. Trees that were partially uprooted, uprooted, snapped, or had canopy damage of >90% were used to estimate mortality. Size classes are as follows: juveniles (<15 cm dbh), small-mature (15–30 cm dbh), medium-mature (30–45 cm dbh), or large-mature (45 + cm dbh).

The wet flatwoods site at St. Marks National Wildlife Refuge (NWR), furthest from the storm center (85 km, Fig. 1), showed the least effect of Hurricane Michael; with no significant change in overall tree density (p = 0.8; Fig. 2, Table 1, Supplementary Figure S1). Mature trees were only represented by the small-mature size class (15–30 cm dbh) at this site (Table 1). Overall estimated mortality, i.e., the ratio of dead to total (living and dead) trees across all represented size classes, was 7.4% (95% CI: 1.3–21.1%) (Fig. 3). There was a significant decrease in tree density in the juvenile size class (p < 0.0001; Table 1, Supplementary Figure S1). There was no significant change in density in the small-mature size class (p > 0.88; Table 1, Supplementary Figure S1). The only documented mortality was in the smallest mature size class (8.7% mortality; CI: 1.9–25.1%), where 4 trees/ha were snapped (Table 2).

**Table 1:** Density assessment of longleaf pine trees pre- and post- Hurricane Michael.

	Pre-Hurricane								Post-Hurricane							
	St. Marks NWR		Joe Budd WMA		Apalachicola NF		Apalachee WMA		St. Marks NWR		Joe Budd WMA		Apalachicola NF		Apalachee WMA	
Juveniles (<15 cm DBH)	22.2	(22.1, 22.3)	13.8	(13.7, 13.9)	11.5	(11.4, 11.6)	13.7	(13.6, 13.8)	13*	(13, 13.1)	13.6*	(13.5, 13.7)	14.2	(14, 14.3)	15.1	(14.9, 15.2)
Small-Mature (15–30 cm DBH)	77.4	(50.18, 119.5)	63	(45.4, 87.4)	31.2	(15.6, 62.7)	15.4	(8.5, 27.8)	81.1	(52.6, 125.2)	76.5	(56.8, 103)	44.7	(25.3, 79.2)	16.5	(6.18, 44.1)
Medium-Mature (30–45 cm DBH)	0.0		127.8	(101.9, 160.3)	27.4	(13.05, 57.4)	70.0	(53.0, 92.3)	0.0		97	(74.8, 125.8)	14.9	(5.6, 39.8)	8.24*	(2.1, 32.9)
Large-Mature (45 + cm DBH)	0.0		10.3	(3.98, 26.4)	0.0		26.6	(13.6, 51.8)	0.0		17.6	(7.8, 39.6)	0.0		5.85*	(1.3, 26.7)
Total Mature Tree Density	77.5	(50.5, 118.8)	202.8	(169.5, 242.7)	58.6	(35.4, 97.3)	116.1	(93.7, 144)	81.2	(52.9, 124.5)	194.0	(161.5, 233.1)	59.7	(36.6, 97.5)	33.0*	(16.5, 65.9)
Total Living Tree Density	103.3	(71.3, 149.6)	219.9	(185, 261.3)	78.2	(50.4, 121.2)	135.7	(111.2, 165.6)	96.6	(65.3, 143)	211.0	(177, 251.6)	82.1	(54.1, 124.7)	53.6*	(31.1, 92.2)

Density estimates are reported in trees·ha-1 followed by 95% confidence intervals in parentheses. Post-hurricane densities with a significant decrease from pre-hurricane densities at p-value <0.05 are noted with an asterisk. The apparent increases in the mature size classes at some sites were not statistically significant (p > 0.05; Supplementary Figure S1).

**Table 2:** Damage Classification.

	St. Marks NWR		Joe Budd WMA		Apalachicola NF		Apalachee WMA	
No Visible Damage	55	(93.3%)	127	(82.5%)	27	(50.0%)	12	(8.3%)
Minor	0		19	(12.3%)	18	(33.3%)	4	(2.8%)
Partially Uprooted*	0		0		2	(3.7%)	6	(4.1%)
Uprooted*	0		0		7	(13.0%)	46	(31.7%)
Snapped*	4	(6.7%)	7	(4.5%)	0		70	(48.3%)
Canopy Loss >50%	0		1	(0.6%)	0		1	(0.7%)
Canopy Loss >75%	0		0		0		3	(2.1%)
Canopy Loss >90%*	0		0		0		3	(2.1%)

The damage classification included both living and dead trees. Values are reported in trees·ha^−1^ followed by the total percentage from each site. Trees were classified as follows: no visible damage, minor damage (minor visible damage such as needle loss or fallen branches), partially uprooted, uprooted, snapped, or minor to major crown damage including canopy loss of >50%, >75%, or >90%. *Trees that were partially uprooted, uprooted, snapped, or have canopy loss of 90% were considered dead.

The sandhill forest at Joe Budd Wildlife Management Area (WMA) was the next furthest site from the storm center (56 km, Fig. 1). This site had no significant changes in tree densities pre- and post-hurricane (p > 0.05; Fig. 2, Table 1, Supplementary Figure S1), except in the juvenile size class (p < 0.001). This site had the highest overall tree density and all size classes were represented (Table 1). Overall estimated mortality was 4.6% (95% CI: 1.9–9.1%) (Fig. 3). The apparent increase in density in the small-mature size class was not statistically significant (p = 0.38; Table 1, Supplementary Figure S1). All the trees that were observed to have died were in the medium-mature size class (30–45 cm dbh) and snapped rather than uprooted (Table 2). Mortality in the medium-mature size class was 9.5% (95% CI: 4.1–18.6%).

The wet flatwoods site in Apalachicola National Forest (NF) was closer, only 35 km from the hurricane path (Fig. 1). The overall living tree densities were again similar and had no significant change from pre- to post-hurricane (p = 0.87; Table 1, Fig. 2, Supplementary Figure S1). This site had a higher overall tree density than St. Marks NWR and had trees in all size classes except the largest size class (45 + cm dbh). Overall mortality was 15.4% (95% CI: 5.1–32.2%) (Fig. 3). The apparent increases in density overall, overall mature, and in the small-mature size class were not statistically significant (p = 0.87, 0.96, 0.43, respectively; Table 1, Supplementary Figure S1). Mortality across size classes showed greater mortality in the mature size classes; up to 14.3% (95% CI: 3.1–38.5%) in the small-mature size class and 33.3% (95% CI: 7.7–71.9) in the medium-mature size class (Fig. 3). All trees that died were uprooted or partially uprooted (Table 2).

**Figure 4:**
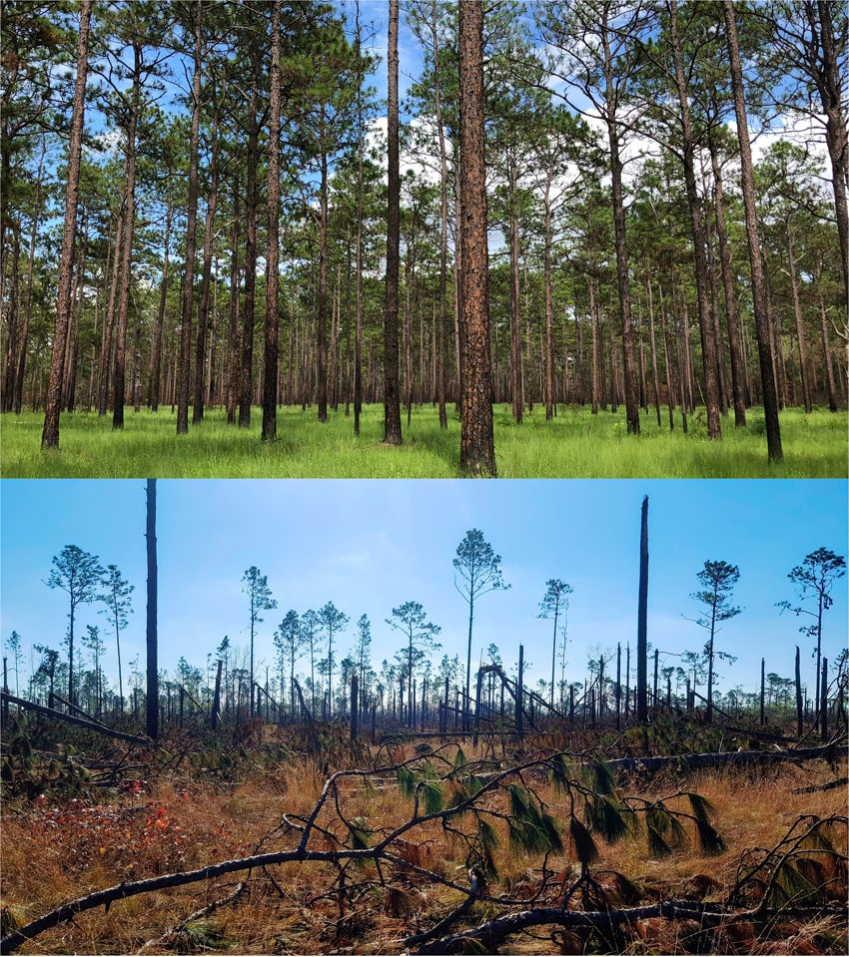
Apalachee WMA. Pre-hurricane, July 7^th^, 2018 (top, image: C. Anderson) and post-hurricane, December 1^st^, 2018 (bottom, image: N. Zampieri).

Finally, the Apalachee Wildlife Management Area (WMA) site was directly in the path of Hurricane Michael (2 km, Fig. 1) and easily the most severely impacted (Fig. 4). All size classes were represented at this site (Table 1). Medium, large, overall, and overall mature size classes showed significant decreases in living tree densities (p = 0.003, 0.04, 0.002, and 0.007, respectively; Fig. 2, Table 1, Supplementary Figure S1). The apparent increase in density in the small-mature size class was not statistically significant (p = 0.9; Table 1, Supplementary Figure S1). Overall mortality was estimated at 87.8% (95% CI: 80.8–93.1%) (Fig. 3). Mortality was highest in the medium-mature size class at 97.6% (95% CI: 92.4–99.5%), followed by small-mature trees at 75% (95% CI: 50.9–90.9%), then large-mature trees at 50% (95% CI: 12.3–87.7%) (Fig. 3). Almost all trees at this site had some visible damage and most dead trees were snapped (48.3%) (Table 2).

## Discussion

The Florida Panhandle is a stronghold for the longleaf pine ecosystem, with more connected, protected longleaf pine habitat than anywhere else in its range^37,38^. Considering that the total range of longleaf pine habitat is estimated at 1.9 million ha^63^, our results show that 28% and 6% of all remaining “known” longleaf pine habitat experienced tropical storm and hurricane force winds, respectively, in Florida alone. Understanding the extent of habitat impacted by this one extreme storm highlights the importance of conserving large and connected areas since varying tree mortality, degrees of habitat integrity, and vulnerability to storm damage exist within this range. While the storm had an extremely wide breadth (560 km diameter), the greatest impact of the storm was highly localized to the area immediately in the path of the storm center. The majority of impacted habitat likely benefitted from the low tree mortality and subsequent opening of canopy gaps. However, for the region within the path of the storm center, longleaf pine habitats were severely impacted. Our results show that longleaf mortality ranged from 4.6–87.8% across sites and medium sized trees (30–45 cm dbh) were the most vulnerable due to snapping (Fig. 3, Table 2).

Since longleaf pines are the dominant and often the only canopy species in these systems, their mortality is necessary for creating canopy gaps^30,34^ that promote the unique biodiversity in this habitat^8,30,34^. Currently, lightning is considered to be the primary cause of mortality in longleaf pines, estimated to be 0.29–0.46 trees ha^−1^ year^−1^^64,65^, and therefore is seen as the main driver of gap dynamics^64–66^. Platt and Rathbun (1992) recognized that the rate of mortality due to hurricanes exceeded that of lightning strikes when considered over a longer timeframe (e.g., 10 years). Our study shows mortality between 4–125 trees ha^−1^ (Table 2), 14–425 times higher, occurring during just one extreme event in one concentrated area. Furthermore, trees with minor damage or canopy damage may experience delayed mortality due to storm related injuries^5,34^. In the Florida Panhandle alone, there have been 10 major hurricanes to make landfall since 1851^67^. Given the average return interval for a hurricane in the Florida Panhandle of 9–13 years^41^, or 1 major hurricane every 2 years for the entire U.S. coastline^41^, it is possible that historically hurricanes may have played a more important role in maintaining gap dynamics of longleaf pines than lightning at longer temporal scales. However, lightning kills individual trees, which promotes heterogeneity across the landscape^64^, whereas hurricanes impact large swaths of the landscape, creating a more homogenous effect, especially near the strongest part of the storm. After Hurricane Hugo (Category 4, 1989), second-growth stands of longleaf in South Carolina experienced 95% adult tree mortality^68^. Hurricane Kate (Category 3, 1985) resulted in over 20% mortality of adult longleaf from an old-growth stand, with effects continuing for at least 5 years post-hurricane^34^. These strong storms represent the degree of impact that is predicted to increase with current anthropogenic climate change.

The four sites surveyed in this study represent two distinct community types, which, in addition to distance to the storm center, may have played a role in the type of tree damage caused by high wind events. Wet flatwoods (WF) and upland pine (UP) communities differ in species composition and structure, hydrology, and soil type^18^. WF sites are inundated for parts of the year, and the water table is relatively close to the surface^18^. In contrast, upland pine sites are dry, well drained, and have a greater distance between the water table and the surface. Trees in wet or mesic habitats develop a shorter taproot and may be more likely to be uprooted in high wind events^69^, whereas in xeric habitats trees develop a longer taproot which makes them more likely to snap or have crown damage^18,34,69^. At the WF site in Apalachicola NF, all dead trees were uprooted or partially uprooted. In the case of St. Marks NWR, the other WF site, all dead trees were snapped (4 trees ha^−1^), though it was also the furthest from the storm center (85 km) and experienced low mortality overall. At the UP sites, as expected, trees were more likely to be snapped than uprooted. All the trees that died at Joe Budd WMA (56 km away) were snapped and at Apalachee WMA the most common damage type was snapping (48.3%), followed by uprooting (31.7%).

In general, and especially at Apalachee WMA, mortality increased with size from juvenile to mature size classes, and then decreased in the largest mature size class when those size classes were present (Fig. 3). The medium-mature size class was the most represented in this study, and thus had a greater exposure risk than other size classes. However, we expected lower mortality in the juvenile size class because the juvenile wood of longleaf pine trees, with a high proportion of living sapwood to heartwood, is more elastic than mature wood, providing greater resistance to high wind^51,70^. Our results are consistent with other studies, such as in an assessment of hurricane-induced mortality of longleaf pines in South Carolina where lower mortality (<20%) in juvenile-younger mature size classes was found in comparison to larger mature size classes, which had up to 95% mortality^71^. At an old-growth stand of longleaf pines in Georgia and at a stand of south Florida slash pine (*Pinus elliottii* var. *densa*) hurricane induced mortality rates were also higher in the larger size classes^34,72^.

The significant loss of mature trees reduces the current extent of mature habitat, on which many critically endangered species depend^17^. High mortality in the medium and large mature size classes can have important implications for the recovery of the system because the larger trees produce the most reproductive cones^34^ and produce the most leaf litter, an important component of the ecology of fuels^60^. Large-mature trees (>45 cm dbh) had lower rates of mortality than medium-mature trees when present – as much as 50% lower (Apalachee WMA, Fig. 3). However, this size class was the least represented in our study, only found at the UP sites and in low quantities. Another explanation for the lower rate of mortality in the large-mature class could be that since these trees are generally fewer in number than other size classes, the surviving individuals could have traits that have enabled their survival thus far and therefore are more resilient to high winds (e.g. a deeper taproot, fewer lower branches contributing to structural imbalance, or differences in wood density)^55,73–75^. More study is needed to determine if large-mature trees are in fact less vulnerable to strong storms.

While the remaining juveniles could represent the potential for recovery, this depends on managing potential pests and invasive species and reintroducing fire quickly, which may require substantial efforts to remove fallen trees and debris. Active fire management will be critical to restoration in longleaf pine habitats affected by Hurricane Michael and future extreme storm events^21–23,60,76^. Reintroducing fire soon after such a concentrated mortality event will aid in the reduction of woody plants that are benefiting from the reduced competition and canopy gaps^60^. In all instances where trees were killed, by snapping or uprooting, the increased biomass on the ground contributes to fuels for fire and at a fine-scale change fire behavior by creating microsites that burn at hotter temperatures for longer amounts of time^77,78^. In order to reintroduce fire to some of the more heavily damaged sites, low impact timber salvage may be necessary to remove dangerous fuel sources, reduce smoke emissions, and open up the understory to promote fire contiguity while minimizing risk of severe wildfire and impact to the soil and understory^71,79^. In the site that experienced the highest rates of mortality, where the mature trees were severely reduced (Fig. 4), restoration may require planting of seedlings as natural regeneration may be impeded by the disruption to the seed bank and ability to manage with prescribed fire due to debris and negative impacts of salvage logging^43,60,68^. Even then, recovery could take decades for juveniles to reach mature size classes (Fig. 4). These changes in the ecology of fuels will have significant ramifications for the long-term maintenance of biodiversity, structural diversity, and the recovery of the system^60^.

## Conclusion

In the NACP, storms of increasing strength and frequency pose a significant threat to the longleaf pine ecosystem and the numerous species that depend on it. Here we take advantage of a fortuitous census of four sites before Hurricane Michael struck North Florida then create a Before-After study of hurricane effects in longleaf pine habitats in the direct path of the hurricane. We show that Hurricane Michael resulted in varying rates of mortality on longleaf pines in the Florida Panhandle with the most severe impact highly localized to the center of the storm and resulting in catastrophic losses of mature canopy trees (up to 98%). This study focuses on the impact of Hurricane Michael in Florida, but the storm impacted most states within the NACP, all containing critical longleaf pine habitat. The increasing frequency of extreme stochastic events requires updating restoration and management plans for critical habitats^7^. The remaining extent of longleaf pine ecosystems exist in varying degrees of habitat integrity^37^ and even protected high quality habitat is ecologically vulnerable to climate change. Moving forward, we must consider the implications of changing disturbance regimes due to anthropogenic climate change on the ecology of critical habitats.

## Methods

### Hurricane coverage and extent of impacted habitat

Data on the storm track and wind extent was obtained from the National Hurricane Center. Hurricane force winds extended outward from the storm center for 75 km and tropical storm force winds extended 280 km^62^. Using ArcMap 10.6.1, we created buffers around the storm track for hurricane and tropical storm force winds. We then overlaid the buffers on longleaf pine habitat coverage within Florida obtained from the Longleaf Pine Ecosystem Geodatabase (LPEGDB) (https://www.fnai.org/longleafgdb.cfm). The LPEGDB is a publicly available geodatabase managed by the Florida Natural Areas Inventory (FNAI) with extensive data on the distribution and ecological condition of longleaf pine habitat in Florida. FNAI identified pinelands using aerial images, data provided by agencies, field surveys, and parcel data. Pinelands were then classified by longleaf pine occurrence as “known”, “expected”, “potential”, or “pinelands other than longleaf”. According to FNAI, “known” habitat has been confirmed through field surveys, “expected” are expected to be longleaf dominated based on historical vouchers, natural community type, and/or presence of red-cockaded woodpeckers, and “potential” are identified as having a community type that may be suitable for longleaf but there are no records of presence and further assessment is needed^37,39^. We then extracted the area of known, expected, and potential longleaf habitat within the hurricane force and tropical storm force wind buffers to determine the known and potential extent of habitat impacted by the storm within Florida.

### Site description

In the summer of 2018, pre-Hurricane Michael, we surveyed several ‘exemplary’ longleaf pine reference sites^80^ throughout the state of Florida to assess longleaf pine density, age and size structure. Four of these initially surveyed sites were in the path of Hurricane Michael and are the focus of the Before-After assessment in this study. FNAI selected individual sites to serve as natural community reference sites based on canopy structure, regeneration, and overall groundcover quality (https://www.fnai.org/RefNC_Playlist_map/index.html). The longleaf pine community reference sites are well managed (with active fire management), exemplary representations of their respective community types and are mostly comprised of second-growth stands of naturally occurring longleaf pine^37,80^.

The four sites in this study represent two different natural community types, wet flatwoods (WF) and upland pine (UP), ranging between 2 and 85 km away from the center of the storm, where it was the strongest (Fig. 1). The two WF sites were in St. Marks National Wildlife Refuge (NWR) (85 km from center of storm) and Apalachicola National Forest (NF) (35 km from center of storm). The two UP sites were in Joe Budd Wildlife Management Area (WMA) (56 km from center of storm) and Apalachee Wildlife Management Area (WMA) (2 km from center of storm). Three of the four sites were located on the eastern side of the storm while the fourth site (Apalachee WMA) was located directly within the center of the storm. We were not able to access sites on the western side post-storm.

### Pre- and post-hurricane field surveys

Prior to the hurricane, sites were fortuitously surveyed in April and May of 2018 for assessing stand structure. Field surveys of tree density, life-stage, and size structure were conducted using modified variable area transects, where distances between trees and a baseline transect were used to estimate density^81^. A baseline transect was extended 40 meters and divided into 8 cells (4 on each side, each 10 m wide and variable in length) to make a plot. Within each cell, data on the closest 5 living trees were recorded, including GPS location, diameter at breast height (dbh), and distance to the furthest tree from the baseline transect, for a maximum of 5 trees per cell or a maximum search distance of 20 m per cell. We chose the modified variable area transects because variable-area transects allow for appropriately large sample sizes^82^, particularly in widely spaced longleaf pine savannas, and we used the modification by Sheil *et al*.^81^, which produces density estimates for different species, to produce density estimates for different size classes in this study. The number of plots varied from 2–5 depending on the size of the stand, to capture a representative sample of each site. Plot locations captured a representative sample of each site by equally spacing plots throughout the sites, which varied from 2.1–24.2 ha (Supplementary Table S1). Trees were classified into 4 possible size classes based on their life stage and dbh: juveniles (<15 cm dbh) and mature trees (with evidence of cone production) that were small (15–30 cm dbh), medium (30–45 cm dbh), or large (45 + cm dbh). In pre-hurricane surveys, dead trees were infrequently encountered and were not recorded because the goal of these surveys was to determine living tree density.

Post-hurricane surveys were conducted in November and December of 2018, within 3 months of the storm, using the same variable area transect methodology^81^. Plot placements in post-hurricane surveys were determined using GPS coordinates taken at the starting point of the original (pre-hurricane plots), using high resolution maps of pre-hurricane surveys, and following the same cardinal direction of original plots. Although plot placement matching prior surveys was not exact, the variable-area transects are designed to capture representative density estimates for the site. During post-hurricane surveys, additional information was recorded, including the status of the tree (living or dead) and any visible damage. Post-hurricane surveys were conducted two ways. First, a survey of remaining living trees was conducted for the Before-After assessment of tree density. Second, a survey of all trees (living and dead) was conducted to determine the density of dead trees as well as percent mortality. In addition to all living trees, only trees killed by the storm were included in post-hurricane surveys, determined through a visual assessment of tree decay. Trees killed by other causes were infrequently encountered and had signs of decay inconsistent with recent storm damage (e.g., significant levels of rot, no green foliage remaining, or had evidence of experiencing a fire post-mortem. There were no fires between the hurricane and our post-hurricane surveys). Living and dead trees were classified into the following damage groups: no visible damage, minor damage (such as needle loss, broken, or fallen branches), partially uprooted, uprooted, snapped, or moderate to major crown damage, for which estimated percent canopy loss was also recorded (canopy loss of >50%, >75%, or >90%). Canopy loss of 75% or greater included damage to the main stem and majority needle loss. Canopy loss of 90% included damage to the main stem and total needle loss. Trees that were partially uprooted, uprooted, or snapped or had canopy loss of >90% were considered dead. Trees with canopy loss of >90% comprised only <1% of the sample and removing them from mortality estimates do not substantially change our results.

### Statistical analysis

We quantified the effects of the hurricane on tree density in two ways. First, we compared densities of living trees in pre- and post-hurricane surveys, and second, we directly estimated mortality by comparing the density of living and dead trees post-hurricane. For the former, we estimated densities of pre- and post-hurricane trees by size class using generalized linear mixed effects models, where site and the interaction between site and survey (i.e., before vs. after) were fixed parameters, the count of trees per size class per cell was the response, and sample plot within site was the random effect. The models were weighted by the area searched in each cell (following the survey design by Sheil *et al*.^81^). Plots were used as the random effect because not every size class was represented in every cell. We used generalized linear mixed models with a Poisson likelihood from the lme4 package^83^ in R. We estimated marginal means and confidence intervals with the emmeans package^84^ to determine if changes in density from pre- to -post-hurricane surveys were significant at the p < 0.05 level. Second, we used a logistic regression to estimate mean longleaf pine mortality overall and of each size class at each site. The response was the count of living trees by size class over the total number of trees (living and dead) observed per cell by size class with a categorical fixed effect capturing site number and size class, weighted by the total number of trees in each size class per cell. We generated 95% confidence intervals for mortality with a Jeffrey’s interval method^85^.

## Supplementary information

Supplementary Information.

## Data Availability

The datasets generated during and/or analysed during the current study are available from the corresponding author on reasonable request.
