# Supplementary material for: The impact of Hurricane Michael on longleaf pine habitats in Florida
Source: Sci Rep. 2020 May 21;10:8483. doi: 10.1038/s41598-020-65436-9 (PMC7242371; doi:10.1038/s41598-020-65436-9)
Supplement: Supplementary file 1 — Supplementary Information. [file 41598_2020_65436_MOESM1_ESM.pdf]

# Supplement

# The impact of Hurricane Michael on longleaf pine habitats in Florida

Nicole E. Zampieri[1*], Stephanie Pau[1], Daniel K. Okamoto[2]

[1]Department of Geography, Florida State University, 113 Collegiate Loop, Tallahassee, 32306, FL, USA
[2]Department of Biological Science, Florida State University, Tallahassee, 32306, FL, USA
[*]Corresponding author
E-mail: nz13@my.fsu.edu (NZ)

**Supplementary Figure S1.**

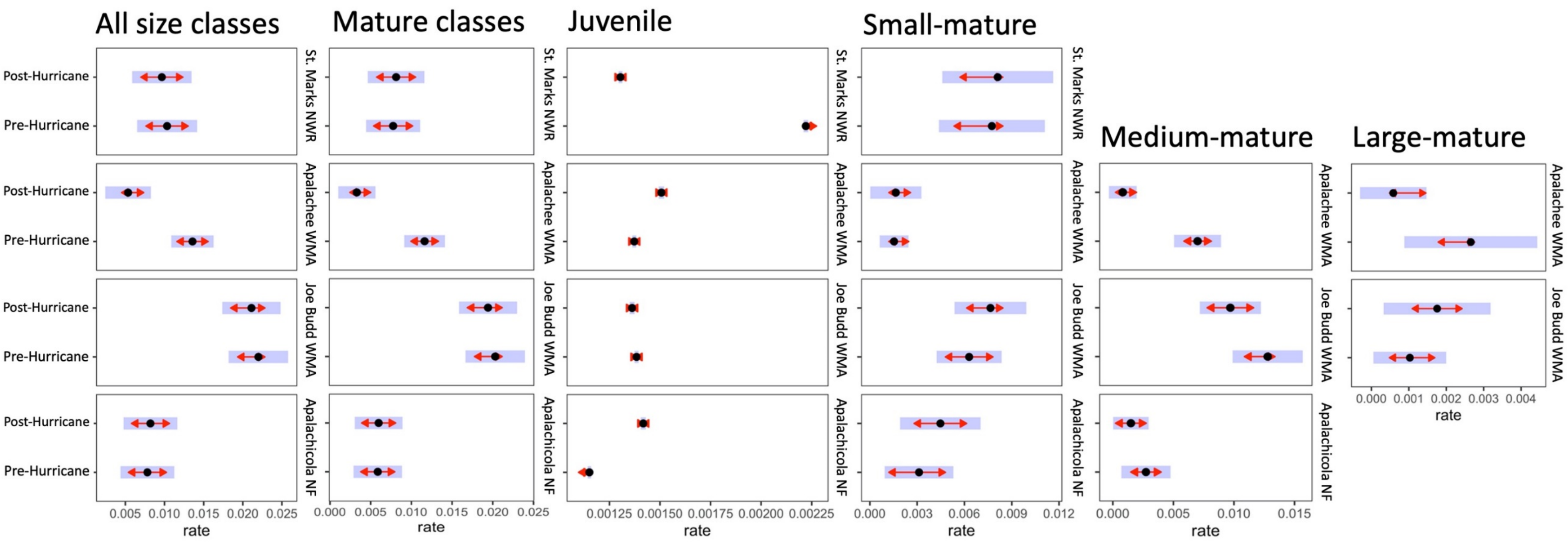


Rates are the estimated marginal mean density in trees·ha$^{-1}$ on the log scale for all size classes, overall mature size classes, and individual size classes by site. Blue bars indicate confidence intervals for mean densities and red arrows are pairwise comparisons among them. Red arrows that do not overlap indicate significance at the $p < 0.05$ level. These were used to determine significant decreases in pre- and post-hurricane densities in Table 1. Size classes are as follows: juveniles (<15 cm dbh), small-mature (15-30 cm dbh), medium-mature (30-45 cm dbh), or large-mature (45+ cm dbh).

**Supplementary Table S1**. Site names, type, size, and number of plots used at each.

| Site Name | Community Type | Hectares | Plots |
|---|---|---|---|
| St. Marks National Wildlife Refuge (NWR) | Wet Flatwoods | 3.33 | 2 |
| Joe Budd Wildlife Management Area (WMA) | Upland Pine | 10.98 | 4 |
| Apalachicola National Forest (NF) | Wet Flatwoods | 2.14 | 2 |
| Apalachee Wildlife Management Area (WMA) | Upland Pine | 24.19 | 5 |

Sites were selected from the existing list of FNAI reference sites and varied in size from 2.14 - 24.19 ha. Number of plots were determined by site size. The same number and location of plots were used in pre- and post-hurricane surveys.
